# Mutation breeding of high-stress resistant strains for succinic acid production from corn straw

**DOI:** 10.1007/s00253-024-13112-7

**Published:** 2024-04-01

**Authors:** Jing Wu, Yilian Li, Jinbao Yin, Chen Wang, Xuejin Qi, Yujie Zhou, Hongjuan Liu, Pengfei Wu, Jianan Zhang

**Affiliations:** 1 Shanxi Key Laboratory of Chemical Product Engineering, College of Chemical Engineering and Technology, Taiyuan University of Technology, Taiyuan, 030024 China; 2 Institute of Nuclear and New Energy Technology, Tsinghua University, Beijing, 100084 China; 3 College of Life Science and Technology, Yangtze Normal University, Fuling Chongqing, 408100 China

**Keywords:** Succinic acid, *Actinobacillus succinogenes*, Atmospheric room temperature plasma, Corn straw, Fermentation

## Abstract

**Abstract:**

The production of succinic acid from corn stover is a promising and sustainable route; however, during the pretreatment stage, byproducts such as organic acids, furan-based compounds, and phenolic compounds generated from corn stover inhibit the microbial fermentation process. Selecting strains that are resistant to stress and utilizing nondetoxified corn stover hydrolysate as a feedstock for succinic acid production could be effective. In this study, *A. succinogenes* CICC11014 was selected as the original strain, and the stress-resistant strain *A. succinogenes* M4 was obtained by atmospheric and room temperature plasma (ARTP) mutagenesis and further screening. Compared to the original strain, *A. succinogenes* M4 exhibited a twofold increase in stress resistance and a 113% increase in succinic acid production when hydrolysate was used as the substrate. By conducting whole-genome resequencing of *A. succinogenes* M4 and comparing it with the original strain, four nonsynonymous gene mutations and two upstream regions with base losses were identified.

**Key points:**

*• A high-stress-resistant strain A. succinogenes M4 was obtained by ARTP mutation*

*•  The production of succinic acid increased by 113%*

*• The mutated genes of A. succinogenes M4 were detected and analyzed*

**Graphical Abstract:**

**Figure:**
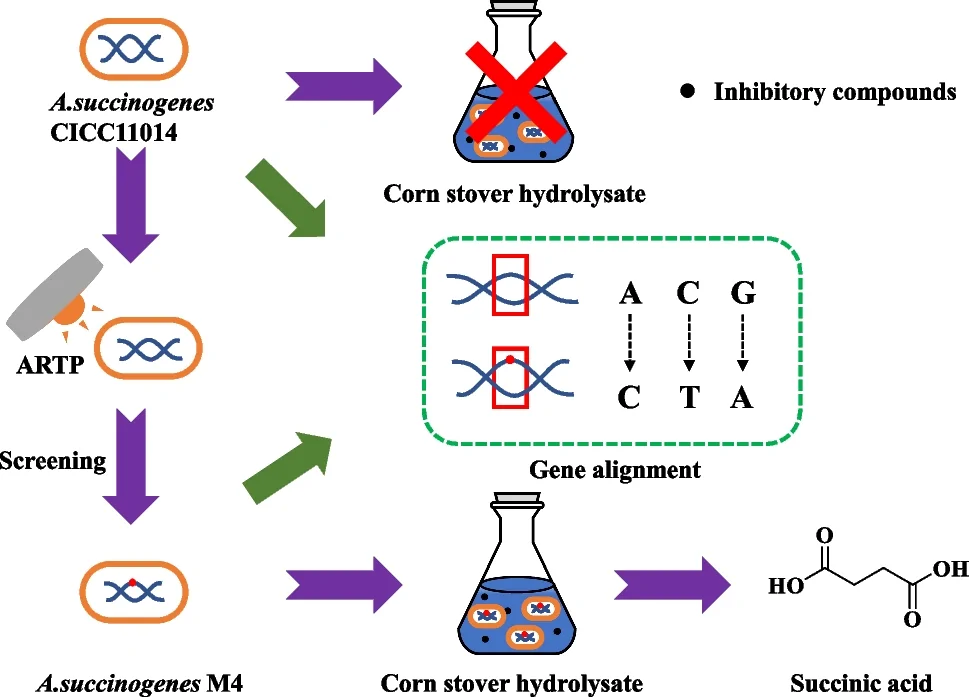

## Introduction

Succinic acid is a four-carbon platform chemical widely used in various fields, such as chemical, food, pharmaceutical, and cosmetics. It is considered one of the most valuable biobased products for the future (Bozell and Petersen 2010; Willke and Vorlop 2004). Succinic acid is mainly used in the food and pharmaceutical industries as an acidifier, pH adjuster, and antibacterial agent, as well as in the production of antibiotics, vitamins, and amino acids (Li et al. 2021; Prabhu et al. 2020). Traditional succinic acid is produced from fossil fuels, but its production capacity is limited (Platnieks et al. 2020). The current major demand for succinic acid is in the production of 1,4-butanediol, which in combination with succinic acid is used to produce polybutylene succinate (PBS). Research shows that 4% of the world’s production of bioplastics is from PBS (Thakur et al. 2022). According to international market analysis, the market of biobased SA was estimated at $175.7 million in 2017 and is forecasted to reach $900 million by 2026, with a compound annual growth rate (CAGR) of 20% (Jokodola et al. 2022). However, the current production capacity for succinic acid falls short of international market demand and provides ample growth opportunities. Microbial fermentation with renewable resources for succinic acid production, a sustainable green approach, has great potential for the development of biobased succinic acid (Narisetty et al. 2022; Xu et al. 2023).

Corn straw is one of the most widely used biomass materials and contains cellulose and hemicellulose that can be degraded into monosaccharides under certain conditions to provide a carbon source for microbial growth and fermentation (Shi et al. 2015). The main characteristic of producing succinic acid from lignocellulosic materials is that the hydrolysate contains both pentose and hexose sugars. *A. succinogenes* has the advantage of a broad substrate range and can ferment a variety of carbon sources to produce succinic acid, making it a suitable choice for utilizing lignocellulose (Lu et al. 2021; Zheng et al. 2009). However, due to the complex structure of straw, the efficiency of succinic acid fermentation from such material is lower than that from sugar and starch materials; thus, pretreatment is required to convert the raw material into monosaccharides before it can be used by microorganisms (Li et al. 2022; Wang et al. 2019). Pretreatment of corn stalks with dilute acids can produce several inhibitory compounds, such as organic acids, furans, and phenolic compounds, which seriously affect microbial growth and fermentation (Wu et al. 2013). Therefore, detoxification is necessary to remove these inhibitory substances from the hydrolysate before fermentation. However, detoxification processes can increase production costs due to the loss of fermentable sugars, the need for additional chemical reagents, and the use of toxic reagents in some detoxification methods (Jonsson et al. 2013; Li and Chen 2008). Selecting strains that display resistance to inhibitory substances and fermenting the hydrolysate directly can significantly reduce production costs.

*A. succinogenes* is capable of fermenting multiple sugars from hydrolysate to produce succinic acid. However, its tolerance to inhibitors is limited (Dessie et al. 2018). Mutation breeding can be used to screen stress-resistant strains effectively. Traditional mutagenesis techniques, such as ultraviolet radiation, X-rays, sodium azide, and diethyl sulfate, are inefficient and harmful to humans. Atmospheric and room temperature plasma (ARTP) mutagenesis is a novel method that generates high-activity plasma jets of various active particles (e.g., excited helium, oxygen, nitrogen atoms, and hydroxyl radicals) at temperatures between 25 and 40 °C and at ambient pressure (Zhang et al. 2014a, b). ARTP is characterized by multiple active particle types, simple operation, mild conditions, a fast mutagenesis rate, and high safety. It has already been successfully applied to the mutagenesis breeding of bacteria, fungi, yeast, actinomycetes, and microalgae (Cao et al. 2017; Liu et al. 2015; Zhang et al. 2014a, b). Qi et al. used *Rhodosporidium toruloides* as the parental strain and screened for a strain with improved inhibitor tolerance using ARTP mutagenesis. This strain was able to produce lipids by fermenting nondetoxified hydrolysate (Qi et al. 2014). Our group used ARTP mutagenesis to obtain a mutant strain of *Enterobacter cloacae* with twofold improved tolerance to dilute acid hydrolysate from corn cobs. The mutant strain was able to produce 2, 3-butanediol by fermenting nondetoxified hydrolysate (Wu et al. 2019). In this study, *A. succinogenes* CICC11014 was used as the parental strain, and ARTP mutagenesis was applied to obtain mutant strains with improved inhibitor tolerance. The mutant strain was able to produce succinic acid using nondetoxified corn straw hydrolysate as a substrate. Then the genome of the mutant strain was sequenced and compared to that of the parental strain. The mutation sites were identified and the mechanism by which the mutant strain exhibited improved inhibitor tolerance was analyzed. The present work provides a good strain for producing succinic acid from lignocellulosic biomass and serves as a theoretical basis for the genetic engineering of strains with improved inhibitor tolerance.

## Materials and methods

### Bacteria and reagents

The strain used in this study was *Actinobacillus succinogenes* CICC11014 (*Actinobacillus succinogenes* 130Z ATCC55618), which was purchased from the China Center of Industrial Culture Collection (CICC). The sequence accession number of the genome sequence is CP000746.1. Corn straw (30–60 mesh) was purchased from Jiangsu Lianyungang Surui Straw Processing Plant, China. The cellulose, hemicellulose, and lignin contents of the corn straw were 33.1%, 23.2%, and 22.2%, respectively. Cellulase (3000 IU/g) and xylanase (10,000 IU/g) were purchased from Hunan Youtel Biochemical Co., Ltd., China.

### Medium

The activated culture medium (TSA) consisted of 17 g/L tryptone, 3 g/L soy peptone, 5 g/L sodium chloride, 2.5 g/L potassium hydrogen phosphate, 2.5 g/L glucose, and 15 g/L agar. The seed culture medium (TSB) consisted of 17 g/L tryptone, 3 g/L soy peptone, 5 g/L sodium chloride, 2.5 g/L potassium hydrogen phosphate, and 2.5 g/L glucose. The primary screening media used were 75% diluted acid hydrolysate, 15 g/L yeast powder, and 15 g/L agar. The fermentation medium consisted of 30 g/L glucose, 4.2 g/L disodium hydrogen phosphate, 1.3 g/L potassium dihydrogen phosphate, 15 g/L yeast powder, 4.6 g/L magnesium chloride, 0.5 g/L sodium chloride, and 0.1 g/L calcium chloride. The fermentation medium of hydrolysate was straw dilute acid hydrolysate containing 15 g/L yeast powder.

### Preparation of dilute acid hydrolysate

Corn stover and dilute acid solution were mixed in a solid–liquid ratio of 1:3 (w/w) and transferred to a conical flask. The mixture was subjected to pretreatment at 145 °C, an acid concentration of 0.97%, and 30 min. Following pretreatment, solid–liquid separation was achieved using vacuum filtration. During filtration, hot distilled water at a temperature of 60 °C, equivalent to twice the mass of corn stover, was used for washing to obtain additional sugar. The resulting hydrolysate was adjusted to pH 6.5 using Ca(OH)_2_ and filtered to obtain the dilute acid hydrolysate.

### Seed culture

The *A. succinogenes* strain was stored in a glycerol vial at – 80 °C and inoculated into solid activation medium and statically cultured at 37 °C for 18–24 h. A single colony of the activated strain was then selected and inoculated into a 200-mL anaerobic bottle containing 50 mL of seed culture medium. The system was made anaerobic by introducing 100% CO_2_ into the bottle, which was then incubated in a shaker at 37 °C and 120 rpm for 14–16 h.

### Fermentation experiment

The seed culture was transferred into a 200-mL anaerobic bottle containing 50 mL fermentation medium and magnesium carbonate. The addition amount of magnesium carbonate was 65% of the total sugar concentration. The system was again anaerobic and incubated on a shaker at 37 °C and 120 rpm. The inoculation amount of pure sugar fermentation medium was 5%, and the inoculation amount of hydrolysate fermentation medium was 10%.

The fermentation tank experiment was conducted in a 2 L fermenter containing 1 L of fermentation medium with a glucose concentration of 60 g/L. The fermentation conditions included a CO_2_ aeration rate of 0.2 vvm, a stirring speed of 200 rpm, and a temperature of 37 °C. The pH was controlled at 6.5 using saturated sodium bicarbonate. The inoculation amount was 5%.

### ARTP mutagenesis

The ARTP mutation process was performed according to previous methods (Wu et al. 2019). The device was operated with helium gas as the working gas at a flow rate of 10 L/min and an output power of 120 W. The original *A. succinogenes* strain was inoculated in the seed medium and cultured until the OD_600_ reached 0.8–1.0. After that, 10 μL of the bacterial solution was removed and placed on a stainless steel sheet, which was subsequently placed on the sample stage. Different plasma treatment times were applied to induce mutations in the bacterial cells. The stainless steel sheet was then transferred to 1 mL of TSB for rinsing, and the culture medium containing the mutagenized cells was diluted to different concentrations. These dilutions were subsequently spread onto TSA and primary screening media and incubated at 37 °C for 24 h. The bacterial count and mortality rate were calculated as follows: mortality rate (%) = (*W* − *M*)/*W*, where *W* is the number of colonies on the TSA plate before mutagenesis treatment and *M* is the number of colonies on the TSA plate after mutagenesis treatment.

### Continuous passage culture

The mutant strains were inoculated into a 200-mL anaerobic bottle containing 50 mL of seed culture medium and then incubated in a shaker for 16 h at 37 °C and 120 rpm. The grown culture was subsequently used to inoculate another anaerobic bottle at 5% inoculum size, repeating the above procedure. Before inoculation, all the anaerobic bottles were infused with 100% carbon dioxide to create an anaerobic environment.

### Analytical methods

The concentrations of substances in the hydrolysate and fermentation process were determined using Shimadzu high-performance liquid chromatography (Japan). Separation of the samples was achieved using an organic acid column (Aminex® HPX-87H, Bio-Red, USA) under the following conditions: a column temperature of 65 °C, a mobile phase of 5 mmol/L H_2_SO_4_, and a flow rate of 0.8 mL/min. Glucose, xylose, arabinose, formic acid, and acetic acid were detected using an RID-10A detector, while furfural and HMF were detected at 280 nm using an SPD-20A detector. An automatic sampler was used to inject 20 μL of the samples into the chromatograph.

### Enzyme activity detection

To prepare crude enzymatic extracts, 30 mL of fermented broth was collected into a 50-mL centrifuge tube and centrifuged for 15 min at 12,000 rpm at 4 ℃. The resultant bacterial pellet was washed twice with 0.1 mol/L Tris–HCl buffer (pH 7.0) and resuspended in 2 mL of buffer (containing 1 mmol/L DTT) before being subjected to ultrasonic homogenization for 15 min at 200 W, with a working frequency of 1 s and an interval of 3 s in an ice bath. The homogenate was then centrifuged at 12,000 rpm at 4 °C for 15 min, after which the supernatant was collected as the crude enzyme extract. The soluble protein content of the crude extract was quantified using the Bradford method (Bradford 1976). The enzyme activity was measured in a reaction volume of 1 mL, where 100 μL of crude extract was added. One enzyme activity unit (U) was defined as the amount of enzyme required to catalyze the oxidation of 1 μmol of NADH or NAD per min at 37 ℃.

The reaction systems used for measuring the activity of various enzymes were as follows (der Werf et al. 1997):

1. The Pepck reaction system: 100 mmol/L Tris–HCl (pH 6.6), 10 mmol/L MgCl_2_, 5 mmol/L MnCl_2_, 75 mmol/L NaHCO_3_, 20 U malate dehydrogenase, 10 mmol/L ADP, 0.3 mmol/L NADH, and 10 mmol/L PEP.
2. The malate dehydrogenase (Mdh) reaction system: 100 mmol/L Tris–HCl (pH 7.2), 0.3 mmol/L NADH, 5 mmol/L OAA, and 2 mmol/L DTT.
3. The pyruvate kinase (Pyk) reaction system: 100 mmol/L Tris–HCl (pH 8.0), 100 mmol/L KCl, 10 mmol/L MnCl2, 2 mmol/L NADH, 60 U lactate dehydrogenase, 30 mmol/L ADP, and 50 mmol/L PEP.
4. The pyruvate formic acid lyase (Pfl) reaction system: 100 mmol/L Mops-KOH (pH 8.0), 0.2 mmol/L CoA, 8 mmol/L DTT, 1 mmol/L NAD^+^, 5 mmol/L L-malate, 2 U of citrate synthase, 20 U of malate dehydrogenase, and 20 mmol/L pyruvate.
5. The acetic acid kinase (Ack) reaction system: 100 mmol/L Tris–HCl (pH 8.1), 10 mmol/L MgCl2, 5 mmol/L PEP, 5 mmol/L ATP, 10 U pyruvate kinase, 8 U lactate dehydrogenase, 0.3 mmol/L NADH, and 20 mmol/L acetate.

### Whole-genome resequencing

Whole-genome resequencing of the strain was completed by Suzhou Jinweizhi Biotechnology Co., Ltd., China.

## Results

### Tolerance of A. succinogenes to hydrolysate inhibitors

In this study, the tolerance of *A. succinogenes* to inhibitors present in the hydrolysate was investigated to provide a reference for further mutation screening. Although *A. succinogenes* has a certain tolerance to inhibitory compounds (Yu et al. 2010), it cannot grow in the presence of high concentrations of inhibitors. Table 1 shows the concentrations of the hydrolysate components, with dilute acid pretreatment reducing hemicellulose to xylose, resulting in a higher xylose concentration (29.1 g/L) and a total sugar concentration (glucose + xylose + arabinose) of 38.5 g/L. Acetic acid was the most abundant inhibitor.

**Table 1:** Concentrations of the components in the dilute acid hydrolysate of corn straw

Glucose (g/L)	Xylose (g/L)	Arabinose (g/L)	Formic acid (g/L)	Acetic acid (g/L)	HMF (g/L)	Furfural (g/L)
4.82	29.1	4.62	0.73	4.40	0.32	1.07

The hydrolysate was diluted to a certain extent for the fermentation of succinic acid. After 48 h of anaerobic fermentation, Table 2 shows that the original strain *A. succinogenes* CICC11014 could not grow in medium containing 100% hydrolysate or in medium containing 75% hydrolysate but grew normally with sugar utilization greater than 90% in medium containing 50% hydrolysate and a medium containing 25% hydrolysate, and the succinic acid production was 9.85 g/L and 5.12 g/L, respectively. The results indicated that the original strain could tolerate a culture medium containing 50% hydrolysate, acetic acid, furfural, and HMF at concentrations of 1.94 g/L, 0.47 g/L, and 0.13 g/L, respectively. Therefore, medium containing 75% hydrolysate was selected as the primary screening medium after ARTP mutagenesis.

**Table 2:** Fermentation results of hydrolysates with different dilution ratios as substrates

Hydrolysate content in the culture medium	Sugar utilization rate	Succinic acid (g/L)	Formic acid (g/L)	Acetic acid (g/L)
100%	-	-	0.61	3.86
75%	-	-	0.46	2.88
50%	93.0%	9.85	0.65	3.33
25%	97.0%	5.12	0.17	1.71

### ARTP mutagenesis and screening of stress-resistant strains

**Fig. 1:**
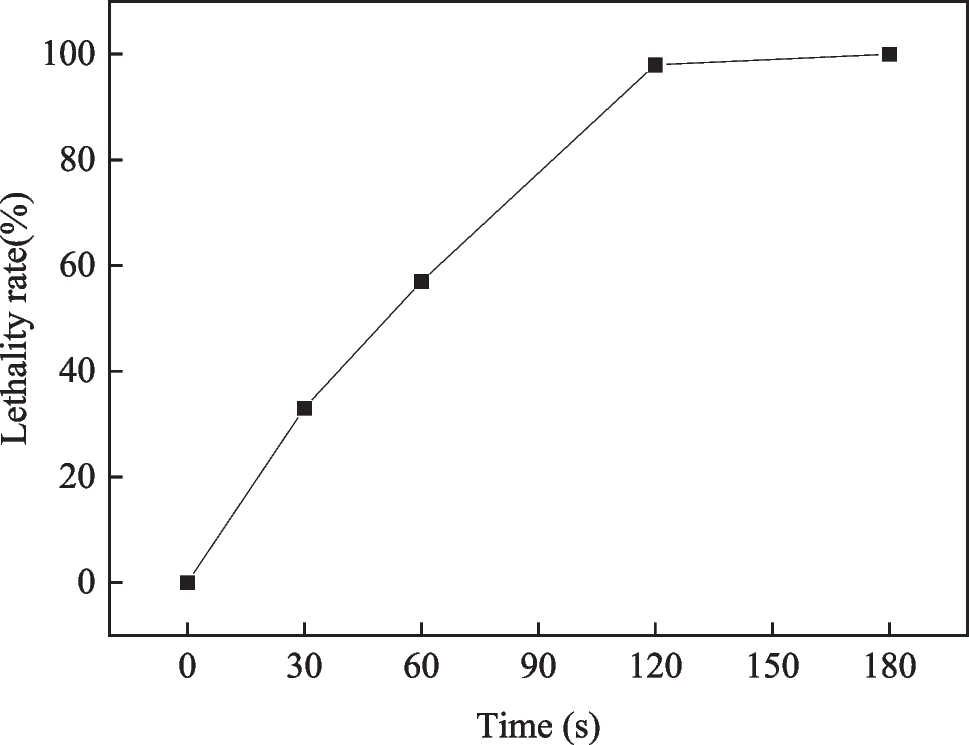
Lethality of cells after different mutation times of ARTP

**Fig. 2:**
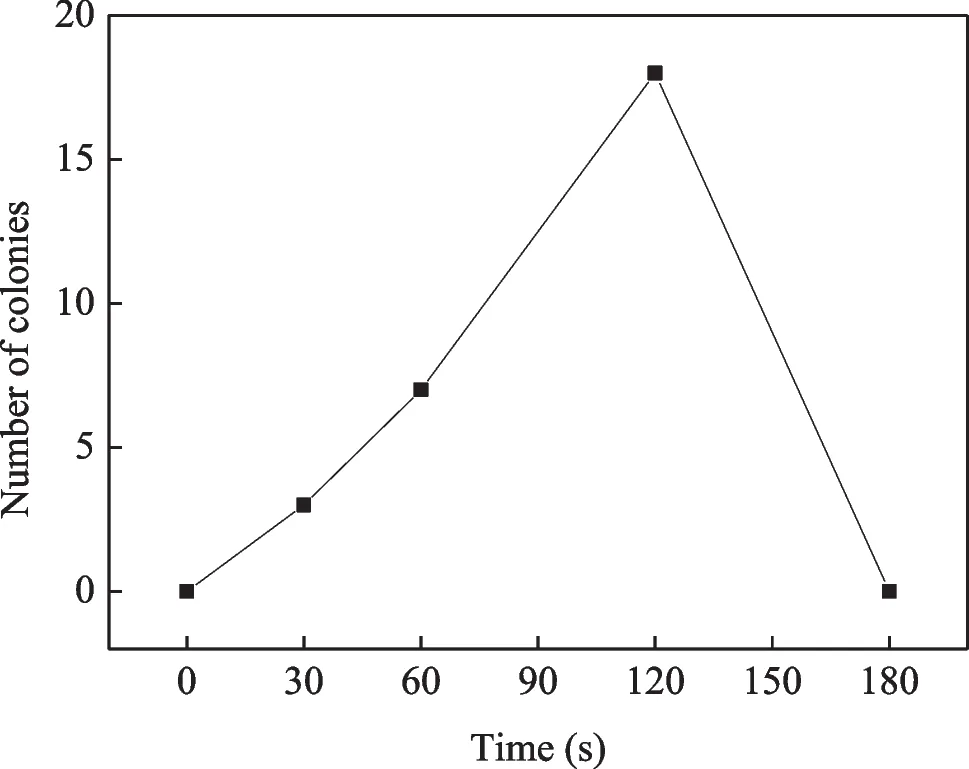
Effect of ARTP mutation time on the number of stress-tolerant bacterial strains

The lethality of bacterial cells under different mutagenic times was investigated. Bacterial solutions were spread on TSA media and primary screening media after mutagenesis for 0, 30, 60, 120, and 180 s. The lethality rates at different mutagenic times are shown in Fig. 1. When the mutagenic time was 30 and 60 s, the lethality rates were low, 33% and 57%, respectively, which was not conducive to further screening. When the mutagenic time increased to 180 s, no colonies grew on the plate, and all the bacteria died. When the mutagenic time was 120 s, the lethality rate was 98%. Typically, bacterial strains that survived after 120 s of mutagenesis were selected for verification via fermentation. However, since hundreds of strains need to be verified, this process is time consuming and labor intensive. Consequently, this study utilized a solid plate with inhibitor-containing hydrolysates as a primary screening medium. The bacterial solutions were simultaneously spread on primary screening media for different mutagenic times. The resulting colonies were resistant to the inhibitors in the hydrolysate. Figure 2 shows the growth of bacterial cells on primary screening media with different mutagenic times. The original strain *A. succinogenes* CICC11014 did not produce any colonies. However, 3, 7, and 18 live bacterial colonies were obtained after mutagenesis for 30, 60, and 120 s, respectively, resulting in a total of 28 strains that were resistant to the inhibitors. Therefore, the optimal mutagenic time was determined to be 120 s with a lethality rate of 98%. The hydrolysate medium containing the inhibitor enables efficient screening of inhibitor-resistant bacterial strains.

**Fig. 3:**
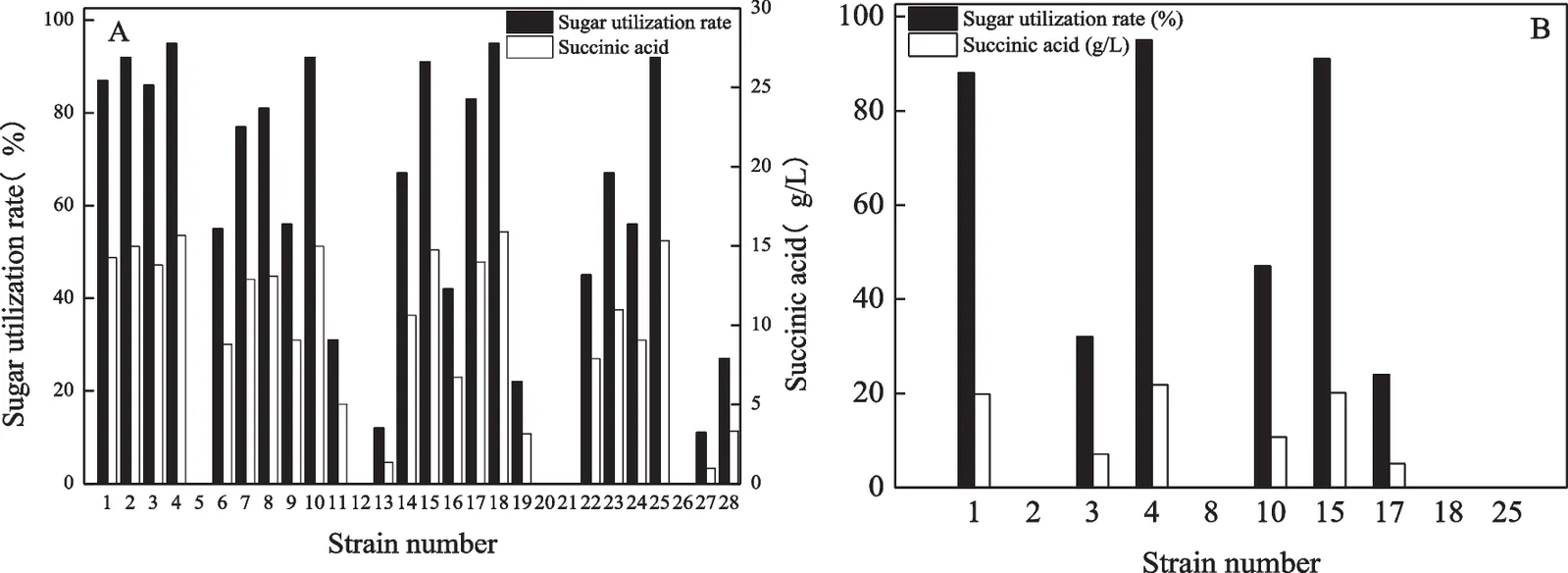
Fermentation performance of the mutant strains using culture media supplemented with hydrolysate, **A** 75% hydrolysate, and **B** 100% hydrolysate

The 28 screened mutant strains were activated and used to produce succinic acid by fermentation using medium containing 75% hydrolysate as a substrate. After inoculation, the initial concentrations of each component in the culture medium were 3.11 g/L glucose, 19.5 g/L xylose, 3.03 g/L arabinose, 2.93 g/L acetic acid, 0.71 g/L furfural, and 0.21 g/L HMF. As shown in Fig. 3A, five of the mutant strains were unable to grow, while 10 exhibited a sugar utilization rate above 80%, with mutant strain 4 demonstrating the highest sugar utilization rate (95%) and producing 15.7 g/L succinic acid. The top 10 mutant strains were subsequently inoculated into culture media supplemented with 100% hydrolysate, and only three strains (1, 4, and 15) exhibited sugar utilization rates above 85% (Fig. 3B), yielding 19.9 g/L, 21.8 g/L, and 20.2 g/L of succinic acid, respectively. Further assessment of mutant strain stability involved five consecutive passages of the three selected strains. The results showed that strain M15 exhibited a significantly reduced fermentation capacity, with a sugar utilization rate of only 45% in undiluted hydrolysate, while strains M1 and M4 had sugar utilization rates of 90.1% and 95.9%, respectively, and produced 18.9 g/L and 21.0 g/L of succinic acid (Table 3). These strains were named *Actinobacillus succinogenes* M1 (M1) and *Actinobacillus succinogenes* M4 (M4), respectively.

**Table 3:** Fermentation results of mutagenic strains after continuous passage

Strain number	Sugar utilization rate (%)	Succinic acid (g/L)	Formic acid (g/L)	Acetic acid (g/L)
M1	90.1 ± 0.65	18.9 ± 0.82	2.65 ± 0.13	6.92 ± 0.62
M4	95.9 ± 0.73	21.0 ± 0.91	2.77 ± 0.07	7.31 ± 0.33
M15	45.0 ± 1.01	9.17 ± 0.55	1.45 ± 0.10	4.78 ± 0.54

### Comparison of fermentation and enzymatic characteristics between stress-resistant strains and the original strain

To compare the fermentation ability of the stress-resistant strains M1 and M4 with that of the original strain, their growth and fermentation characteristics were examined using glucose as the substrate in anaerobic bottles and fermentation tanks. Anaerobic fermentation of approximately 30 g/L glucose for 24 h in bottles yielded results as shown in Table 4. Compared to the original strain, the M1 strain exhibited a 6.6% decrease in succinic acid production and a reduced production of the byproducts formic and acetic acid. M4, which produced 21.4 g/L succinic acid and had a yield of 0.65 g/g glucose, showed a fermentation capacity equivalent to that of the original strain.

**Table 4:** Comparison of the resistance and original strains using glucose shake flask fermentation

Strain	Succinic (g/L)	Formic acid (g/L)	Acetic acid (g/L)	Yield of succinic acid (g/g glucose)
Original strain	20.7 ± 0.54	3.24 ± 0.11	5.15 ± 0.16	0.63
M1	19.3 ± 0.37	2.89 ± 0.14	4.73 ± 0.10	0.60
M4	21.4 ± 0.42	3.55 ± 0.11	5.21 ± 0.13	0.65

**Fig. 4:**
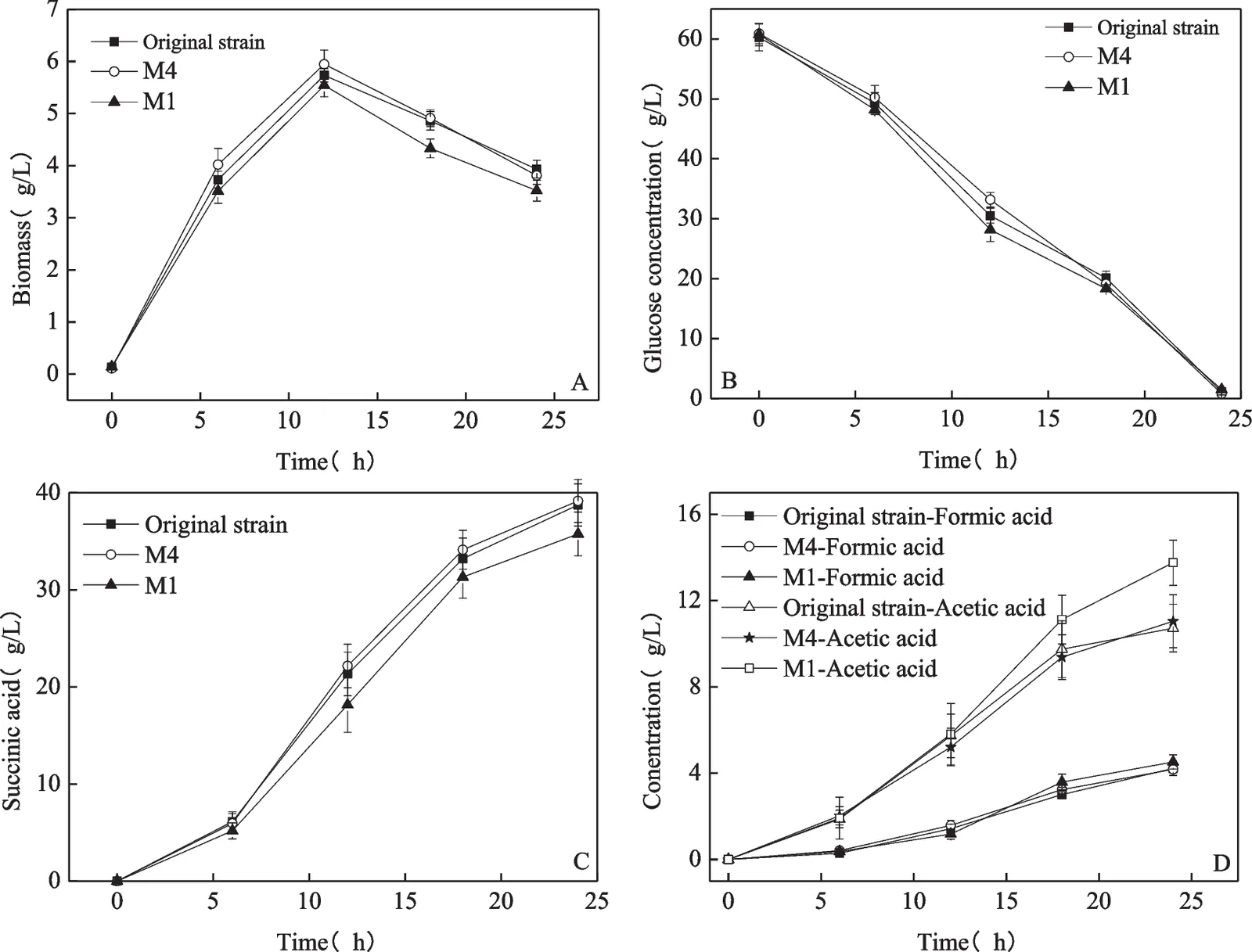
Comparison of fermentation processes between stress-resistant strains and original strains in a fermentation tank

The three strains were cultivated in a fermentation tank to compare fermentation efficiency using 60 g/L glucose as the substrate. Figure 4 shows the changes in biomass, sugar concentration, and product during fermentation. According to Fig. 4A, there was no significant difference in the growth of the three strains during fermentation, and there was no lag phase. The maximum biomass concentration was reached at 12 h, at which time the number of cells gradually decreased as the fermentation time increased. Figure 4B shows that the sugar consumption of the three strains was nearly identical. With respect to the fermentation products, M4 produced succinic acid at a rate comparable to that of the original strain, with succinic acid production reaching 38.8 g/L and 39.2 g/L, respectively, at 24 h, for a yield of 0.65 g/g. The changes in the M1 succinic acid concentration were not significantly different from those of the other two strains within the first 6 h of fermentation; however, during the subsequent 6 h, the succinic acid production of M1 was noticeably lower, and at the end of fermentation, the succinic acid concentration was 35.8 g/L (Fig. 4C). The variations in the byproducts acetate and formate are depicted in Fig. 4D; during fermentation, the formate concentrations did not differ much, with final concentrations of approximately 4.20 g/L. However, M1 produced notably more acetate (13.8 g/L) than did the original strain (10.7 g/L) and M4 (11.0 g/L).

**Fig. 5:**
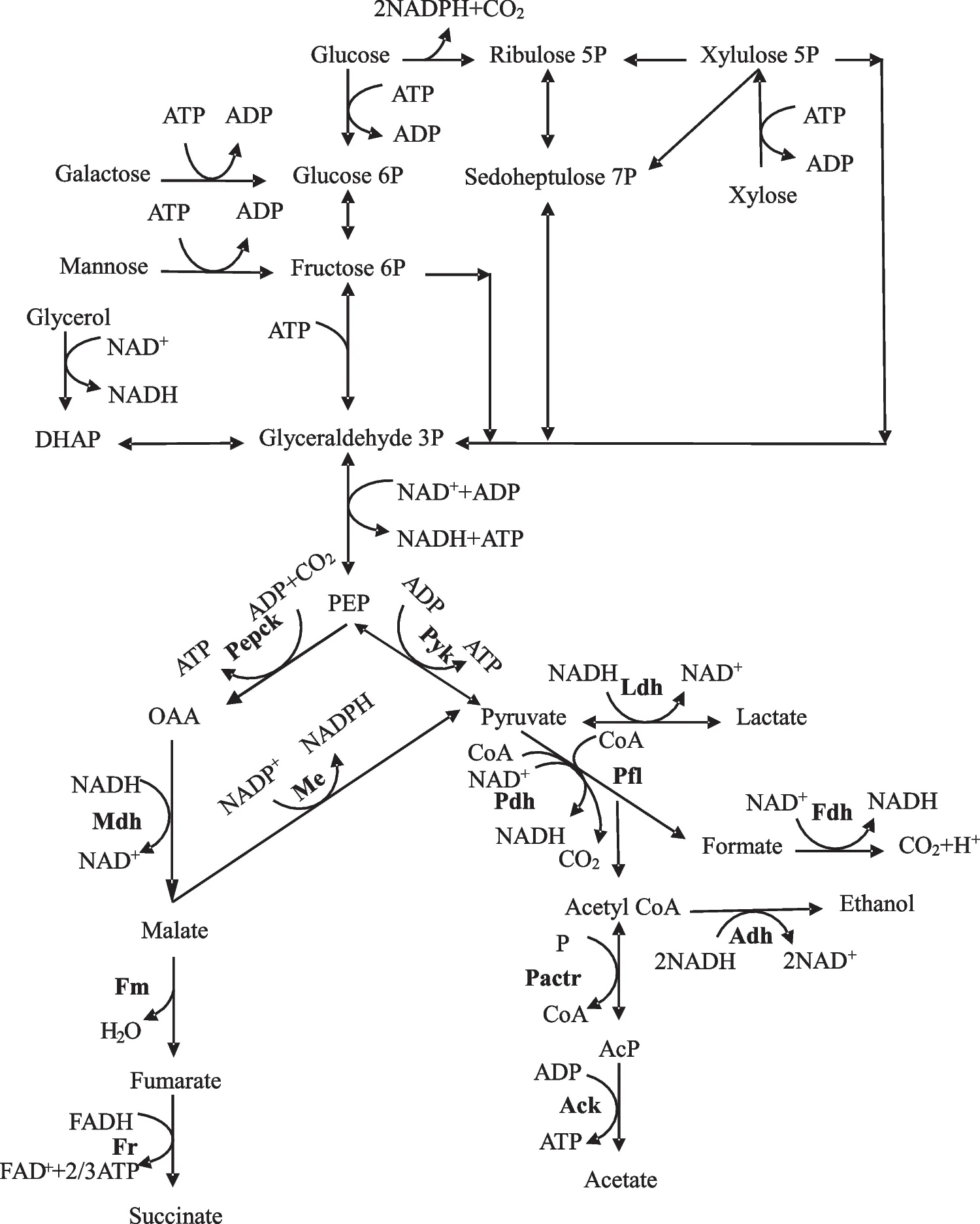
Metabolic pathways of *Actinobacillus succinogenes* associated with succinic acid and its byproducts. AcP, acetyl-phosphate; DHAP, dihydroxyacetone phosphate; OAA, oxaloacetate PEP, phosphoenolpyruvate; Adh, alcohol dehydrogenase; Ack, acetate kinase; Fdh, formate dehydrogenase; Fm, fumarase; Fr, Fumarate reductase; Ldh, lactate dehydrogenase; Mdh, malate dehydrogenase; Me, malic enzyme; Pactr, phosphoryl acetyltransferase; Pepck, phospoenolpyruvate carboxykinase; Pfl, pyruvate formate-lyase; Pyk, pyruvate kinase; Pdh**,** pyruvate dehydrogenase

In the metabolic pathway of *A. succinogenes* (Fig. 5), glucose and xylose are initially converted to PEP. The pathway to produce succinate involves the generation of OAA from PEP in the presence of Pepck, followed by entry into the C4 pathway. OAA is further converted to malate by Mdh with NADH as a co-factor and subsequently converted to fumarate by the actions of Fm. Fumarate is catalyzed to succinate by the actions of Fr, which is an NADH-dependent enzyme. Pepck is considered to be the key enzyme for the production of succinate. Additionally, PEP can be converted into pyruvate through the action of Pyk to enter the C3 pathway, which further results in the formation of byproducts. The main byproducts of the fermentation using *Actinobacillus succinogenes* are formic acid and acetic acid. Pyruvate is catalyzed to formate by Pfl. Pyruvate can also be converted to acetate by three enzymes, Pdh, Pactr, and Ack (Pateraki et al. 2016; McKinlay et al. 2007). The key nodes in the metabolic pathway were involved in the conversion of PEP to OAA and pyruvate. This study evaluated the key enzyme activities affecting product production in three strains after 12 h of fermentation. As shown in Table 5, the critical enzyme activity was not significantly different for the stress-resistant strain M4 compared to the original strain. However, the stress-resistant strain M1 showed a 19% lower Pepck enzyme activity than the original strain, which directly affected the carbon flux entering the C4 pathway and subsequently reduced succinic acid production. Moreover, the Pyk and Ack enzyme activities were greater in M1 than in the original strain, with increases of 24% and 13%, respectively. A higher activity of Pyk increased the carbon flux through the C3 pathway, while the Ack-catalyzed reaction led to more acetic acid production. These results are in line with the observed decrease in succinic acid and increase in acetic acid production in M1 compared to those in the original strain. Therefore, *A. succinogenes* M4 is more suitable for fermenting succinic acid using hydrolysates containing inhibitors.

**Table 5:** Key enzyme activities in the succinic acid metabolism pathway

Key enzyme	Enzyme activity (U/mg)		
	Original strain	M1	M4
Pepck	1087 ± 31	876 ± 26	1137 ± 53
Mdh	893 ± 22	826 ± 14	886 ± 18
Pyk	473 ± 32	586 ± 16	491 ± 23
Pfl	298 ± 13	316 ± 25	307 ± 20
Ack	1434 ± 32	1621 ± 27	1468 ± 28

### Analysis of mutant genes in A. succinogenes M4

Whole-genome resequencing of the *A. succinogenes* M4 strain was subsequently performed, after which the results were compared with those of the original *A. succinogenes* CICC11014 strain. Mutated genes in *A. succinogenes* M4 were identified through single-nucleotide variant (SNV) and insertion or deletion (InDel) detection. Five SNV mutations and three indel mutations were detected, all of which were homozygous mutations. No heterozygous mutations were observed (Table 6). There are two synonymous mutations in the SNV. At the 382 bp position in the peptidylprolyl isomerase gene, the nucleotide C mutates to T, resulting in the amino acid remaining leucine before and after the mutation. At the 912 bp position in the β-ketoacyl-ACP synthase I gene, nucleotide A mutates to C, resulting in a synonymous mutation that does not affect the encoded amino acid.

**Table 6:** Analysis of mutant genes in *A. succinogenes* M4

Mutation type	Mutation location	Reference base	Mutated base	Ribbon location	Gene number	Protein
SNV	315,447	A	C	Exonic	ASUC_RS01455	Elongation factor G
SNV	792,274	C	T	Exonic	ASUC_RS03800	Peptidylprolyl isomerase
SNV	951,040	A	C	Exonic	ASUC_RS004615	β-ketoacyl-ACP synthase I
SNV	97,821	G	A	Exonic	ASUC_RS04730	Ribonuclease G
SNV	1,667,663	A	G	Exonic	ASUC_RS07820	N-acetylglucosamine kinase
InDel	1,029,930	T	-	Exonic	ASUC_RS11105	LexA-regulated protein
InDel	1,148,006	A	-	Upstream	ASUC_RS05460	Porin OmpA
InDel	1,971,944	T	-	Upstream, downstream	ASUC_RS09280, ASUC_RS09290	Protein TolB, protein TolR

The SNV mutation includes three nonsynonymous mutations, one of which is a T-to-G transition at base 1616 of the elongation factor G (EF-G) gene, resulting in the substitution of serine with glycine at position 539 in the peptide chain. At base 892 of the N-acetylglucosamine kinase gene, an A-to-G transition results in the substitution of threonine with proline at position 298 in the peptide chain, potentially altering the protein structure and affecting N-acetylglucosamine metabolism. At base 1175 of the ribonuclease G (RNase G) gene, a G-to-A transition leads to the substitution of arginine with histidine at position 392 in the peptide chain.

According to the InDel data, an adenine was missing at position 190 in the coding gene of the LexA regulatory protein. Two mutations were identified in the upstream and downstream regions of the gene. At position 286 upstream of the outer membrane protein A (OmpA) gene, an adenine base was also found to be missing. At position 652 downstream of the tolB gene and 532 upstream of the tolR gene, a thymine base was found missing.

## Discussion

ARTP mutagenesis is an efficient method for obtaining strain mutation libraries and has been widely applied. The ARTP mutagenesis technique utilizes active particles in plasma to alter the permeability of the cell membrane in microorganisms, resulting in genetic damage. The bacterium’s own DNA repair system can repair damage, but severely damaged or poorly repaired bacteria can die. The mutation conditions, particularly the mutation time, are crucial. Zheng et al. used *A. succinogenes* NJ113 as the parental strain to screen acid-resistant strains via ARTP mutation and found that the optimal mutation time was 30 s. The strain obtained, *A. succinogenes* JF1315, showed an 83.33% increase in succinate yield compared to that of the parental strain at pH 5.8. The purpose of Zheng et al.’s work was to screen acid-resistant strains, and the substrate was glycerol (Zheng et al. 2021). In this study, nondetoxified corn stover hydrolysate was used as a substrate, with the aim of selecting strains with high tolerance to inhibitors in the hydrolysate. *A. succinogenes* CICC11014 was used as the parental strain for ARTP mutation, and the optimal mutation time was 120 s, resulting in a cell survival rate of 98%. Furthermore, high-throughput screening methods are particularly important in strain selection, and we have demonstrated that using inhibitor-containing hydrolysate solid media for plate screening is an efficient approach for selecting stress-tolerant strains. The stress-resistant strain *A. succinogenes* M4 was obtained. The stress-resistant strain *A. succinogenes* M4 was capable of growing in culture medium containing 100% hydrolysate and exhibited a twofold increase in stress tolerance, resulting in a 113% improvement in succinic acid production, producing 21.0 g/L compared to the original strain.

The raw genome resequencing data of *A. succinogenes* M4 were deposited in the SRA database under accession number PRJNA998617. After the *A. succinogenes* M4 genome was compared with that of the original strain, mutations were identified in essential genes, including those of EF-G, N-acetylglucosamine (GlcNAc) kinase, RNase G, and the regulatory protein LexA. These genetic mutations may lead to alterations in the corresponding protein structure. EF-G possesses translocase activity and hydrolyzes GTP to move the ribosome one codon down along mRNA, catalyzing the entry of peptidyl-tRNA at the A site to the P site while vacating the A site. EF-G is also involved in ribosome recycling by interacting with ribosome recycling factor (RRF), ensuring the smooth progression of the next round of protein synthesis. Mutations in the EF-G gene can affect peptide chain elongation during mRNA translation, which can further affect protein synthesis (Di et al. 2022). GlcNAc kinase is an enzyme that transfers a phosphate to GlcNAc to generate GlcNAc-6-phosphate, which can be a precursor for glycan synthesis (Umekawa et al. 2022). It is involved in cytoplasmic cell wall recycling events as a step in the peptidoglycan salvage pathway (El-Araby et al. 2023). RNase G is involved in RNA processing and degradation in cells (Schein et al. 2008). LexA is a vital factor in the SOS response of cells and plays a crucial role in DNA damage repair (Sun et al. 2013). Additionally, the upstream region of the OmpA gene and the downstream region of the tolB gene, which is also the upstream region of the tolR gene, underwent base deletions. OmpA mainly functions to maintain the integrity and stability of the extracellular matrix (Smith et al. 2007). Both the tolB and tolR genes belong to the Tol-pal inner membrane system, which plays an essential role in preserving the integrity and stability of cell membranes (Lloubes et al. 2001). The three nonsynonymous mutations and one frameshift deletion in the gene-coding region may impact the corresponding protein structure of EF-G, N-acetylglucosamine kinase, ribonuclease G, and the regulatory protein LexA. Two base deletions in the upstream and downstream regions of the gene have no impact on protein structure or function but may impact gene expression. These mutations are possibly the main reasons for the high tolerance of *A. succinogenes* M4 to inhibitors. The reason for the enhanced stress resistance of *A. succinogenes* deserves further investigation, whether it is due to single-gene mutations or multisite mutations. The identification of the mutation sites in *A. succinogenes* M4 can provide a reference for rational modification of strains.

## Data Availability

All data generated or analyzed during this study are included in this published article.
